# Comparison of dual-dimensional and rectangular wires in terms of space closure and anchorage loss during retraction with miniimplants: A prospective clinical study

**DOI:** 10.34172/joddd.2020.008

**Published:** 2020

**Authors:** Sangeetha Morekonda Gnaneswar, Premkumar Sridhar

**Affiliations:** ^1^Department of Orthodontics, SRM Dental College, SRM University, Ramapuram, Chennai India; ^2^Department of Orthodontics, Tamilnadu Govt. Dental College, Park Town, Chennai India

**Keywords:** Dental implants, friction, orthodontic space closure, orthodontic wires

## Abstract

***Background.*** In sliding mechanics, archwires should slide easily during the retraction of anteriors. Round wires slide well, but the torque control is a significant problem. Rectangular wires produce effective torque expression but pose a challenge to free sliding due to factors like friction and force used to overcome friction, etc. To utilize the properties of both wires, the wire should be bi-dimensional. Dual-dimensional wire is one such wire with different dimensions in the anterior and posterior sections. This study aimed to compare the amount of space closure and anchorage loss of molars between the rectangular and dual-dimensional wire groups during retraction with mini-implants.

***Methods.*** Forty patients were randomly allocated to two groups (n=20). Patients with rectangular wires formed the control group, and those with dual-dimensional wires formed the experimental group. Mini-implants and NiTi coil springs were used for retraction. Model and cephalometric analyses were carried out to calculate the amount of space closure and anchor loss, before and four months after the study. Statistical significance was set at P<0.05.

***Results.*** The average amount of space closure was higher with DDW (3.98 mm) than rectangular wire (3.22 mm). The difference was statistically significant. No significant difference was found with anchorage loss.

***Conclusion.*** DDW can be used as an alternative to rectangular wires during retraction with mini-implants; however, it cannot replace the rectangular wires completely. Anchorage control was effective with both wires.

## Introduction

The success of any treatment lies in choosing the right materials and techniques to bring forth the expected changes, while preserving the rest of the environment. It holds true for any orthodontic treatment, too. Sliding mechanics is commonly employed for space closure in preadjusted edgewise appliance (PEA). We often encounter some form of resistance to sliding with rectangular wires due to factors like friction, binding, notching, the method of ligation, archwire coating, wire deformation, bracket type, etc. Studies on friction reveal that the effective force must increase two folds to overcome the frictional resistance, resulting in a hazardous overload of the anchorage units.^1-3^ Southard et al^4^ claim that if the teeth are free to slide along the archwire, friction between the brackets and the archwires does not increase anchorage loading. To reduce friction clinically, some practitioners prefer the round wires, or they reduce the rectangular wires in the buccal segments to a more rounded cross-section to minimize binding. Bennett et al^5^ concluded that archwire thinning is effective but causes reduced tooth control in the thinned areas. They further reported that selective torque application is more effective, especially in the incisor region.

Hence, it would be ideal to have the characteristics of both rectangular and round wires in an archwire. In 1970, Schudy and Schudy^6^ described the Bi-Metric System. Gianelly^7^ developed the bi-dimensional technique, using brackets with two different slots. Canon^8^ described dual-flex wires with a flexible anterior section. They were usually welded or soldered. Hence, using them for sliding technique was difficult. Wool^9^ introduced the dual-dimensional wire based on this system. The unique feature of this wire was that the anterior portion was rectangular or square in cross-section to affect torque, and the posterior section was round to allow smooth sliding. When DDW was introduced, it was used with intra- and/or inter-maxillary elastics, and the control of the posterior teeth was insufficient. Currently, with the availability of mini-implants, the potential of DDW can fully be expressed.^10^ Yu Li et al^11^ concluded that the bi-dimensional wire system offered stronger torque control for the anterior teeth compared to the conventional method. Most of these studies^12-14^ employed different bracket sizes, wire sizes, slots, or materials for anterior and posterior sections, each with its benefit.

The present study compared two rectangular and dual-dimensional wires with similar dimension in the anterior section and different dimensions in the posterior section. Unlike the wires that were used in previous studies, DDW does not require any manipulation, such as twisting or welding, to produce a bi-dimensional effect. The null hypothesis of our study was that there existed no difference between the wires in the amount of space closure and anchorage loss.

## Methods

The study was undertaken as a prospective single-center parallel-group comparative study. The ethical clearance was obtained from the Institutional Ethics Committee. Of 300 patients reporting to the Department of Orthodontics, 150 were screened for eligibility. The selection criteria included patients with class I malocclusion with proclined upper anteriors and no or minimal crowding in the upper arch with an average overjet of 6‒7 mm (Table 1). Patients with skeletal malocclusion, history of trauma, past or present signs and symptoms of periodontal disease, medically compromised conditions, and those under prolonged medication were excluded from the study. The sample size was calculated using Open Epi, Version 3, and an open-source calculator based on the means and variances of previous studies for comparison of the rate of retraction and anchorage loss.^15-17^ Power analysis was carried out at a confidence interval (two-sided) of 95%, and a power of 80%. Forty-four patients, aged 17‒25 years, were selected, without any gender bias. There were 21 males and 23 females. Informed consent was obtained from all the patients after explaining the potential risks and benefits of the procedures. The subjects were randomly allocated (ratio: 1:1) to two groups (n=22), using random numbers of a computer-generated table from Graph Pad software. Group A (control group) received conventional rectangular wire, 19*25 SS (G&H Orthodontics Franklin, IN, USA) and group B (experimental group) received dual-dimensional wire [Dual Geometry Wires .021*.021*.018 SS (SPEED System Orthodontics, Cambridge Ontario, Canada)]. Two patients in group A and one patient in group B had implant failure in the second month of the study. Another patient in group B missed the follow-up. They were excluded from the study. The final sample size was 20 patients in each group. The treatment plan included extraction of all the first premolars, leveling, and alignment followed by en masse retraction (Pre-adjusted Edgewise Technique, -0.022*0.028 slot MBT prescription with standard 3M metal brackets, 3M Unitek, Monrovia, CA, USA) with mini-screws. The study period was 4 months. All the clinical procedures were performed by one of the authors. The analyses on the models and cephalograms were carried out by a second operator. When in doubt, a third operator was included. Blinding was implemented for the second-level operators and the statistician.

**Table 1:** Demographic data of the subjects

**Characteristics**		**Mean**
**Age**	17‒25 years	18.16
**Gender**	Males (n=21) Females (n=23)	
**Employment** **status**	Students (n=32) Employed (n=8) Unemployed (n=4)	
**Malocclusion**	Skeletal and dental class I	
**Overjet**	6‒7 mm	6.71
**Crowding**	Minimal (0‒4 mm)	2.31

### Clinical Procedures

After the completion of leveling and alignment (with the sequence of wires from 0.016 NiTi, 0.016 SS through 19*25 SS), a lateral cephalogram (T0) was taken by a single technician with the same magnification, using a cephalometric and panoramic radiographic unit (Planmeca Pm 202 Cc Proline) with an L-shaped wire placed in the molar buccal tube on both sides for easy identification.^15^ Mini implants (1.5*8 mm, Dentos, Daegu, Korea) were placed in the buccal cortical region between the second premolars and the first molars of the upper arch. Dual geometry wires and the conventional rectangular stainless-steel wires were engaged in the respective groups. After two weeks, the implants were loaded with NiTi coil spring (G&H Orthodontics Franklin, IN, USA) between the canines (S Hook, G&H Orthodontics Franklin, IN, USA) and the implant heads on both sides. The force produced (150 grams) was measured by Dontrix gauge (Robust, Germany). The patients were reviewed every three weeks. At the end of the study period, the models and lateral cephalograms (T1) were taken following the same procedure as T0.

The primary outcome measure was the amount of space closure, calculated based on the anteroposterior distance between the upper canines and the second premolars at T0 and T1. The secondary outcome measure was the amount of anchorage loss based on the distance the upper first molars moved from T0 to T1.

### Cephalometric evaluation

The anteroposterior distance between the line drawn perpendicular from the palatal plane to the distal surface of the canines and the mesial surface of the second premolars was measured at T0 and T1, and the difference was calculated as the amount of space closure.^16^ Additionally, the horizontal distance between the long axes of canines and the second premolars was measured with reference to the occlusal plane. The horizontal distance from Ptv (pterygoid vertical) to the distal surface of the first molar on both sides was measured to calculate anchorage loss. Molar rotation was calculated using Ba-N plane and S-N plane as reference planes.^17^ The final values were the average of the left and right sides.

### Evaluation of the models

The reference points and planes were marked on the study models (Table 2). The distances between the reference points marked at T0 and T1, on the Ip, from the canine, second premolar and first molar, were calculated as anteroposterior changes. The horizontal distance from the canine cusp, the central pit of premolar and molar teeth to the Ip was measured for transverse changes. Tangents from the distal and mesial surfaces of the permanent first molars projected to the Ip line and the angles formed were measured as rotational changes of the first molar.

**Table 2:** Reference planes and points on the study model

**(a) I.P (Incisive papilla p erpendicular)**	**A perpendicular line drawn anteroposteriorly on the mid-palatine raphe from the labial frenum through the dental midline and the incisive papilla.**
**(b) MID M RT and MID M LT**	A perpendicular line drawn from the mesial pit of the maxillary permanent first molar to the incisive papilla (RT = right and LT = left side).
**(c) MID PM RT and MID PM LT**	A perpendicular line drawn from the central pit of the maxillary second premolar to the incisive papilla
**(d) MID C RT and MID C LT**	A perpendicular line drawn from the canine cusp to the incisive papilla
**(e) M ANG RT and M ANG LT (MES)**	Tangent projected from the incisive papilla perpendicular to the mesial surface of the maxillary first molar
**(f) M ANG RT and M ANG LT (DIS)**	Tangent projected from the incisive papilla perpendicular to the distal surface of the maxillary first molar

### Statistical analysis

The results were tabulated and analyzed with SPSS 16. The data obtained were parametric in nature as per the Shapiro Wilk’s test for normality. Paired-sample t-test was used to compare the results obtained before and after the study period. Independent-sample t-test was used to analyze and compare the individual parameters of the same group. P<0.05 was considered statistically significant.

## Results

### Model analysis

The values represented are mean values of the data. On the right side, the amount of space closure in group A was 3.31 mm, with 4.01 mm in group B. On the left side, the amounts of space closure for groups A and B were 3.37 mm and 4.07 mm, respectively (Table 3). Transversely, in group A, the right side canine moved 0.12 mm while the left canine showed no movement. In group B, the right canine showed no movement but the left side canine moved 0.25 mm. The right side premolars showed more changes than the left side in both groups. The first molars moved 0.125 mm on the right side and 0.25 mm on the left side in group A, whereas in group B, it moved 0.125 mm on both sides (Table 4). The change in molar position in group A was -0.625° on both the right and left sides, while in group B, the right side molar moved -0.125°, and the left molar showed no change (Table 5).

**Table 3:** Model analysis; the total amount of space closure (anteroposterior) between the canines and second premolars

**Parameter**		**Mean in mms**	**P-value** **(two-tailed)**
**N=20**			
**Right Side**	Group A	3.3	0 .001
	Group B	4	0.002
**Left Side**	Group A	3.38	0.005
	Group B	4.08	0.006

Space closure in group B was more than that in group A (approx.0.7 mm). P<0.05 was considered as statistically significant.

**Table 4:** Model analysis; the transverse control for the canine, premolar, and molar teeth from the midline (incisive papilla perpendicular) on the right and left sides

**Parameter**	**Mean changes (mm)**		**P-value (two-tailed)**	
	N=20			
	**Group A**	**Group B**	**Group A**	**Group B**
**MID-C –RT**	0.125	0	0.334	0.351
**MID C –LT**	0	0.25	0.334	0.335
**MID PM RT**	-0.3750	-0.25	0.619	0.619
**MID PM LT**	0.125	0	0.727	0.727
**MID M RT**	-0.125	-0.125	1.0	1.0
**MID M LT**	-0.25	-0.125	0.55	0.55

Minimal transverse changes were observed with the right premolars and molars in both groups, which were statistically insignificant. There was no transverse change in the position of the left canine in group A and the right canine as well as the left premolar in group B. Negative values indicate palatal movement. P<0.05 was considered as statistically significant. (MID = midline-canine, PM = Premolar, M = Molar, RT = right side, LT = left side).

**Table 5:** Model analysis; rotational control of molars (angular measurement of the firstmolar with respect to the midline)

**Parameter**			**Mean change (d egrees)**	**P-value (two-tailed)**
			**N=20**	
**M Ang RT**	DIS	Group A	-0.625	0.108
		Group B	-0.125	0.117
	MES	Group A	-0.625	0.108
		Group B	-0.125	0.117
**M Ang LT**	DIS	Group A	0.625	0.196
		Group B	0	0.217
	MES	Group A	0.6250	0.196
		Group B	0	0.217

On the left side, there was no change in group B and minimal change in group A. On the right side, mean changes were -0.625^◦^ and -0.125^◦^ in groups A and B, respectively. The changes were statistically non-significant. P<0.05 was considered as statistically significant. (M = molar, Ang = angulation, RT = right side, LT = left side).

### Cephalometric analysis

The average amounts of space closure were 3.23 mm and 3.99 mm in groups A and B, respectively. With reference to the long axis, the space closure was 3.49 mm in group A, with 3.9 mm in group B. The mean anchorage loss was 0.12 mm in group A, with 0.09 mm in group B. Group A showed 1º rotation of molars in relation to SN and Ba-N planes, while there was no change in group B. The changes in the premolar angulation were 0° and -0.06° for groups A and B, respectively. The changes in the canine angulations were 6.8° and 7° for groups A and B, respectively (Table 6).

**Table 6:** Cephalometric analysis for the amount of space closure and molar movement (mean values in mm) and changes in the angulation of molars, premolars and canines with reference to SN, Ba-N planes (N-20, mean values in degrees)

**Parameter**	**Group A**			**Group B**		
	**N=20**				
	**T0**	**T1**	**P-value** **(two-**t**ailed)**	T0	T1	**P-value** **(two-**t**ailed)**
**Mes –Dis**	5.65	2.42	0.001	5.96	1.97	0.001
**Long axis**	10.76	7.27	0.001	12.08	8.18	0.001
**Pt V-6**	23.75	23.87	0.850	23.77	23.86	0.229
**SN-Molar**	77.25	76.25	0.170	75.25	75.25	1.00
**Ba-N-Molar**	101.75	100.75	0.121	94.75	94.75	1.00
**Ba-N-Pm**	112.75	112.81	0.844	112.75	112.81	0.844
**Ba-N-Canines**	98.5	105.25	0.001	102.25	109.25	0.001

T0, T1-At the start and end of the study period. Mean amount of space closure was 3.36 mm in group A while it was 3.9 mm in group B, showing 0.5mm more with group B. Molar movement (mesial) was 0.03 mm more in rectangular wire than DGW. The anteroposterior change in molars was very minimal and was statistically insignificant. P<0.05 was considered as statistically significant (Mes-Dis-mesial of premolar and distal of canine). Changes in Molar angulation were about 1◦ with rectangular wires. DDW (DGW) showed no change in molar angulation. Premolars showed similar changes with both the wires. Change in canine angulation was 6.8◦ in group A and 7◦ in group B. Intra group change in canine angulation was significant. All the other changes were statistically insignificant. P<0.05 was considered as statistically significant (Ba = basion, N = nasion, Pm = premolar).

### Intergroup comparison

The anteroposterior measurement between the canine and second premolars showed a significant difference, both on the models (space closure = 13‒15, space closure = 23‒25, P=0.005) and cephalograms (mes-dis distance, P=0.01) (Table 7).

**Table 7:** Comparison of group A and group B (N=20)

**Parameter**	**Group A** **(mean/SD)**	**Group B** **(mean/SD)**	**P-value** **(Two-tailed)**	**T-value**
	Cephalometric Measurements			
**Space closure (Mes-Dis distance)**	3.225/0.477	3.98/0.61	0.01	2.78
**Long Axis**	3.48/0.73	3.9/1.55	0.50	0.68
**Pt V-6**	1.25/1.28	0.27/0.45	0.06	2.02
**SN-6**	0.75/0.71	0.38/0.5	0.24	1.21
**Ba-N-C**	6.8/0.25	7/0.43	0.08	1.7
**Model analysis**				
**Space closure 13‒15**	3.37/0.47	4.0/0.28	0.005	3.27
**Space closure 23‒ 25**	3.37/0.48	4.07/0.36	0.005	3.28
**Mid C Lt**	0.13/0.35	0.13/0.35	1.0	0
**Mid C Rt**	0.13/0.35	0	0.33	0
**Mid Pm Rt**	0.38/0.52	0.25/0.46	0.61	0.5
**Mid Pm Lt**	0.38/0.52	0.13/0.35	0.27	1.12
**Mid Mol ar Lt**	0.25/0.46	0.13/0.35	0.5	0.6
**Mid Molar Rt**	0.63/0.74	0.13/0.35	0.1	1.7
**M Ang Rt**	0.63/0.74	0.13/0.35	0.1	1.7
**M Ang Lt**	1/0.93	0.25/0.46	0.05	2.04

P<0.05 was considered as statistically significant. Total space closure was statistically significant in both cephalometric and model Analyses (Mid = midline, Pm = premolar, Rt = right, Lt = left, Ang = angulation).

## Discussion

Resistance to sliding due to friction is studied under three different phenomena: 1) classic friction; 2) binding; and 3) notching. Classic friction exists when the wire slides without binding and notching. Binding occurs at different stages of orthodontic tooth movement. It increases as angulation increases, at the wire‒bracket interface, adding to whatever friction that might have been present in the absence of binding.^18^ In the present study, as the arch was leveled and aligned prior to the study period, binding due to the difference in bracket positions (leading to an increase in angulation) was minimized for both groups. The wires were regularly checked for any distortion or notching to avoid resistance to sliding. Neither of the wires was altered by twisting or welding. Although stainless steel wire ligation is considered ideal, we opted for elastic ligatures for ease and quickness of the placement and patient comfort.

The results of the present study are consistent with Gianelly’s explanation of “The theories of the bi-dimensional approach—the full-size engagement at the anterior segment can give the utmost play to the pre-torque in these brackets, while the clearance at the buccal segments can facilitate the wire sliding in space closure.”^7^ We could find only case reports and not randomized controlled trials on DDW in the literature. In their two case reports, Danielle et al^14^ used Dynforce wires that had a rectangular cross-section in both the anterior (.021*.025) and posterior segments (018*.022). Both DGW and Dynforce had the same dimension in the anterior segment (.021*.025). This maximized the control of incisor tip and torque. Higher rigidity helped prevent canting of the incisal plane. In contrast, Dynforce, DDW had round posterior cross-section, further reducing the coefficient of friction. As recommended by Nanda,^19^ 0.018-inch wire in the 0.022-inch slot in the posterior section resulted in less friction but provided adequate stiffness, reducing the buckling tendency of the wire during retraction. Power arms allowed the retracting forces to pass close to the center of resistance of the dental arch to produce a bodily movement of anterior teeth, further reducing the dip in the occlusal plane during space closure. Additionally, the round section offered minimal resistance to sliding.

**Figure 1:**
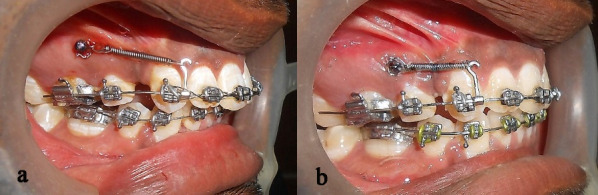
Intraoral right lateral views of a patient in group A with conventional rectangular wire; a and b Right lateral views at T0 and T1, respectively.

**Figure 2:**
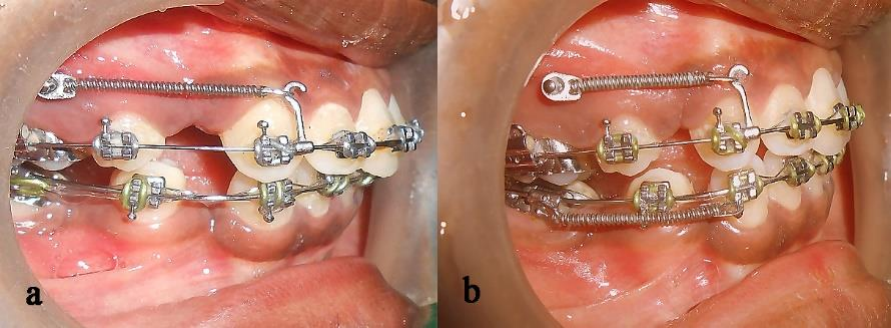
Intraoral right lateral views of a patient in group B with dual-dimensional wire; a and b Right lateral views at T0 and T1, respectively.

In case of the heavier rectangular wire, the surface of the wire contacted the bracket edge with the smallest change in tooth positions due to the low clearance (stiffness) at the bracket‒wire interface. This produced more friction (classic friction and binding) leading to increased resistance to sliding. Hence, space closure was more pronounced in DDW than the rectangular wires, despite a relatively heavier anterior cross-section. The results of the present study showed that the average amount of space closure with rectangular wires was 3.22 mm (Figure 1), with3.98 mm with DDW (Figure 2). Similar results were observed with respect to the long axis parameter. The rate of retraction was calculated at 0.8 mm for the rectangular wire and 0.99 mm for DDW. Our value of the rate of canine retraction (0.8‒1 mm) compares favorably with the values found by Ziegler et al^20^ and that of Thiruvenkatachari et al.^17^ The change in the canine angulation with rectangular wires was less than the DDW by 0.2°, and the difference was statistically insignificant. The difference in the average amount of space closure between the wires was 0.76 mm, which was statistically significant. Hence, the null hypothesis was rejected. We found that patients felt more comfortable with round cross-section in the posteriors than stiffer rectangular section, although this did not affect their routine or that in this study.

Since the mini-implants provided direct anchorage, anchorage loss was minimal for both groups. The results of the present study are consistent with those of a study by Buzzoni et al.,^21^ who showed that a small round wire will minimize binding at the entrance and exit of the bracket, with the partial engagement minimizing the tipping of the molar teeth. Similarly, the small, round posterior section of DDW minimized binding and hence reduced the tipping of molars. Linear movement of the upper first molars with respect to Ptv in group A was 0.12 mm, with 0.09 mm in group B. Changes in the angulation of molars were 0.5‒1° in group A and 0‒0.25° in group B on the average. Rectangular wires showed 0.5‒0.75° more anchorage loss due to increased load on the molars to overcome frictional resistance. The mean transverse and rotational changes were lower with DDW compared to the rectangular wires, and the difference was statistically insignificant. Hence, anchorage control was effective with both wires.

The limitation of this study was that only two wires with similar dimensions were compared for a limited period. Studies with different types of bi-dimensional wires for a longer period would reveal more for a better understanding and application in the future.

## Conclusion

In the age of low-friction systems and mini-implants, dual-dimensional wires can be used as an alternative to rectangular wires during retraction if mini-implants are used for direct anchorage.

## Acknowledgments

The authors would like to thank Dr. Preethi Prabakaran and Dr. Roopa kundavai for assessing the values on the models and cephalograms and Dr. Junaid Mohammed for statistical analysis.

## Authors’ Contributions

PKS and SMG: conception or design of the work; SMG and PKS: acquisition, analysis, or interpretation of data for the work; SMG and PKS: drafting the work or revising it critically for important intellectual content. All authors have read and approved the final manuscript.

## Funding

Not applicable.

## Competing Interests

The authors declare no competing interests with regards to the authorship and/or publication of this article.

## Ethics Approval

The protocol of the study was approved by the Institutional Ethics Committee (ref. no. 0430/DE/2010).
